# CoVidAffect, real-time monitoring of mood variations following the COVID-19 outbreak in Spain

**DOI:** 10.1038/s41597-020-00700-1

**Published:** 2020-10-20

**Authors:** Carlos Bailon, Carmen Goicoechea, Oresti Banos, Miguel Damas, Hector Pomares, Angel Correa, Daniel Sanabria, Pandelis Perakakis

**Affiliations:** 1 Research Center for Information and Communication Technologies (CITIC), University of Granada, Granada, Spain; 2 Mind, Brain and Behavior Research Center (CIMCYC), University of Granada, Granada, Spain; 3 Department of Social, Organisational, and Differential Psychology, Complutense University of Madrid, Madrid, Spain

**Keywords:** Databases, Human behaviour

## Abstract

The COVID-19 outbreak and the ensuing confinement measures are expected to bear a significant psychological impact on the affected populations. To date, all available studies designed to investigate the psychological effects of this unprecedented global crisis are based on cross-sectional surveys that do not capture emotional variations over time. Here, we present the data from CoVidAffect, a nationwide citizen science project aimed to provide longitudinal data of mood changes following the COVID-19 outbreak in the spanish territory. Spain is among the most affected countries by the pandemic, with one of the most restrictive and prolonged lockdowns worldwide. The project also collected a baseline of demographic and socioeconomic data. These data can be further analyzed to quantify emotional responses to specific measures and policies, and to understand the effect of context variables on psychological resilience. Importantly, to our knowledge this is the first dataset that offers the opportunity to study the behavior of emotion dynamics in a prolonged lockdown situation.

## Abstract (summary)

**Table:** 

Measurement(s)	Emotion • arousal domain measurement • mood change measurement
Technology Type(s)	questionnaire
Factor Type(s)	emotion dynamics during lockdown
Sample Characteristic – Organism	Homo sapiens
Sample Characteristic – Location	Kingdom of Spain

Machine-accessible metadata file describing the reported data: 10.6084/m9.figshare.12948677

## Background & Summary

On March 11th, 2020, the World Health Organisation (WHO) characterised the new coronavirus disease called COVID-19 as a pandemic^1,2^. In an attempt to restrict the spread of the disease, governments around the world adopted unprecedented confinement measures that had an immediate effect on many people’s usual activities, routines or livelihoods^3,4^. Spain promptly became one of the most affected european countries, counting 9,785 diagnosed cases and 136 COVID-related deaths in early March^5^ (cumulative count before lockdown, on 03/14/20), escalating to 239,429 diagnosed cases and 27,117 deaths by the end of May^5^. On March 14th, the spanish government imposed a widespread lockdown aimed to minimize social contact and avoid the collapse of the national health system^6^. The lockdown measures implemented, which rank among the most restrictive and prolonged worldwide, consisted in closing schools and universities, drastically reducing the population’s mobility, and interrupting all non-essential industrial activity countrywide^7^.

The confinement measures and the COVID-19 sanitary crisis itself led to dramatic changes in people’s behavior and lifestyle, whose potential negative psychological impact was promptly recognised^8,9^. In public mental health terms, levels of anxiety, stress and depression are expected to rise^10^. Accurate information on the population’s emotional response to ongoing events is hence important to best anticipate the needs for psychosocial support and for evidence-based policy making. Since the COVID-19 worldwide outbreak, several studies have attempted to address this important issue by gathering data on psychological and emotional well-being^8,11,12^. All of these studies employed cross-sectional surveys, which capture a static description of the population’s emotional experience. However, research on affect dynamics has shown that identifying the specific pattern of variations in feelings, moods and emotions over prolonged periods of time may be critical for understanding and predicting psychological adjustment and well-being^13^. As a striking example, evidence from diverse research paradigms suggests that mood and anxiety disorders (e.g., depression, bipolar disorder) may be identified by differences in the dynamics of affective experience^14^.

In order to provide longitudinal, openly available, geolocalized data of mood variations in the Spanish territory, we initiated a citizen science project called CoVidAffect. The project comprises the collection and curation of a database of individual changes in subjective feeling (valence) and physical activation (arousal) during the COVID-19 lockdown in Spain. Participants countrywide regularly reported these two fundamental dimensions of emotion via the project’s website or through a smartphone app, developed specifically for this purpose. By means of this methodology, the project aimed to offer longitudinal data to track mood dynamics during the COVID-19 crisis and its different confinement stages, instead of static impressions provided by one-shot questionnaires.

We have monitored mood variations between March 28th, 2020 and June 21th, 2020, when the nationwide state of alarm was lifted, thus allowing social contact and unrestricted mobility. It is important to note that the de-escalation from the initial strict lockdown towards the “new normality” stage, was carried out gradually in well defined phases that can be contrasted with the longitudinal data of our study to investigate the possible effects of different measures and policies on the emotional well-being of the population. As the lockdown was bound to have a different impact on each participant, depending on their particular context, we have also collected contextual information, such as socioeconomic status, living space, employment changes and physical activity levels. The dataset we describe and publish here, includes participants’ daily mood variations, initial contextual information, and weekly reports of changes in context variables. This is, to our knowledge, the first dataset which longitudinally tracks mood variations during the COVID-19 lockdown. The data provided can be used to investigate aspects of the psychological impact of the COVID-19 crisis on the affected population. Interested researchers, organisations and authorities may explore various ways to exploit these data to quantify the population’s emotional response to specific measures and policies, and to understand the effect of certain context variables on emotional regulation and psychological resilience. Importantly, to our knowledge this is the first dataset that offers the opportunity to study the behavior of affect dynamics in a lockdown situation.

## Methods

### Participant onboarding

Since its official release on March 28th 2020, the project has been broadcasted in social media and the Spanish national press. Volunteer participants joined by accessing our website (https://covidaffect.info), which described the scope of the project, offered information about privacy policy, displayed regularly updated news about the project, and presented a summary of the data through an interactive map. No exclusion criteria were applied.

**Fig. 1:**
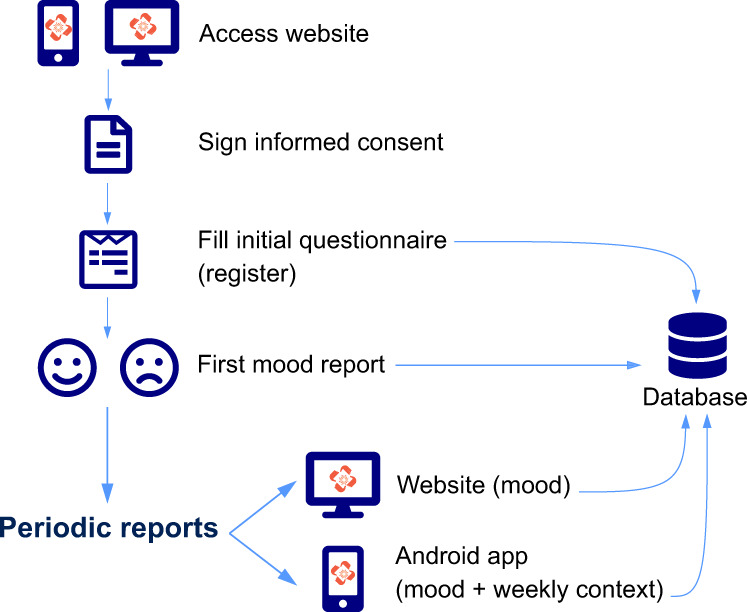
Participant onboarding process.

When participants clicked on the “participate” button at the website, they were asked to fill an intake questionnaire on demographic, context and COVID-19-related data. In order to submit the questionnaire, participants had to sign an informed consent with detailed information about the study, including risks and benefits, privacy protection, and participation rights. After completing the questionnaire and signing the informed consent, they received a unique ID number, which was used as the only means of identification in the subsequent mood assessments. Following the registration process, participants were asked to complete their first mood rating and were encouraged to return and update their mood state frequently. They were also offered the possibility to download the CoVidAffect Android app, which sent regular reminder notifications and allowed the participants to rate their mood directly from the app. A layout of the participants’ onboarding process is provided in Fig. 1.

**Fig. 2:**
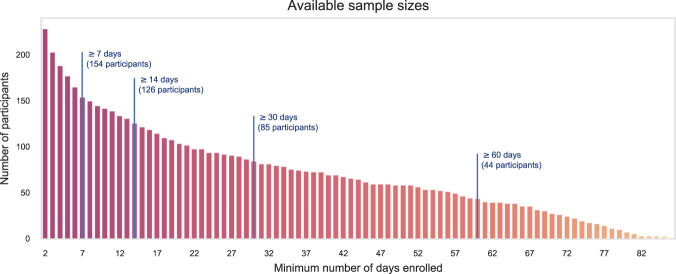
Available sample sizes for longitudinal analysis. For visualization purposes, participants with only one day of enrollment are not included in this figure.

A total of 999 participants successfully registered and reported at least one mood questionnaire. 312 participants (31%) downloaded the app and 687 (69%) reported their mood through the website. The average enrollment length was 23 days, and participants reported an average of 68 mood assessments during the study duration. Figure 2 illustrates the available sample size as a function of enrollment duration. We note that data from 154 participants are available for a time window spanning one week, while at the other end of the spectrum, 44 participants provided data for 60 consecutive days.

### Ethical approval

This project was reviewed and approved by the Human Research Ethics Committee of the University of Granada (ref.: 1378/CEIH/2020).

### Data collection

**Fig. 3:**
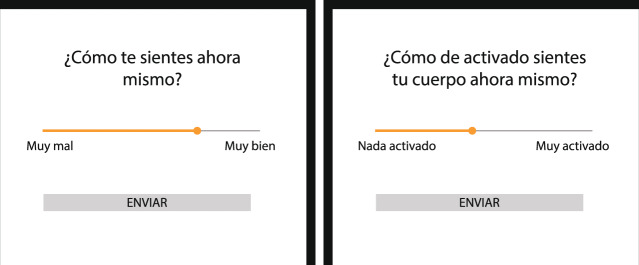
Valence and arousal rating screens triggered by the CoVidAffect smartphone app. English: *‘How do you feel right now?’ (‘Very bad’ - ‘Very good), ‘How physically active do you feel right now?’ (‘Not active’ - ‘Very active’)*.

Participants accessed the project website and used their ID number to rate their subjective feeling and physical arousal as often as they considered necessary and up to 6 times per day. They were also asked to enter their postcode, which was cross-referenced with the ID number to ensure data reliability. In addition, Android smartphone users could download the CoVidAffect app, available on the website. The app has been deployed using a smartphone-based platform for continuous mood monitoring during daily life^15^, by means of Experience Sampling Methods (ESM)^16^. The app triggered the mood assessment questionnaire at pseudo-random times during the following six, evenly distributed one-hour intervals: 07:00–08:00, 10:00–11:00, 13:00–14:00, 16:00–17:00, 19:00–20:00, and 22:00–23:00. Participants received a notification indicating that a new questionnaire was available. Once they tapped the notification, the app triggered the questionnaire and the mood rating screen was displayed (Fig. 3). The app sent the mood ratings, trigger and response timestamps, and participant number to the data storage server. The notifications persisted during a time period of one hour. If they were not answered during that interval, they were automatically dismissed in order to avoid questionnaire overlap. In addition, once a week, app users received a batch of supplementary questions related to current health and lifestyle status, with the aim of gathering information about contextual changes during that week. The content, periodicity, and number of records of the questionnaires are summarized in Table 1.

**Table 1:** Summary of each questionnaire and number of records gathered.

Questionnaire	Trigger frequency	Content	Unique participants	Unique records
Intake	Once, when the participant registers at the website	15 questions including demographic, situational, socioeconomic and contextual information (Table 2).	999	999
Mood	Web users: each time they enter the website App users: six times per day	2 questions rating subjective feeling and physical arousal (Table 3).	999	17,452
Context	Weekly, only available for app users.	8 questions about contextual changes during the week (Table 4).	99	395

**Table 2:** Intake questionnaire.

Question	Response options
Gender	One of {‘Male’, ‘Female’, ‘Other’}
Age	Integer (>= 16)
Postcode	Integer
How many people live with you?	One of {‘I live alone’, ‘1 person’, ‘2 people’, ‘3 people’, ‘4 people’, ‘5 people’, ‘6 or more people’}
Age and relationship of people living with you^a^	Age: integerRelationship: one of {‘Parent’, ‘Spouse/Couple’, ‘Child’, ‘Sibling’, ‘Grandparent’, ‘Grandchild’, ‘Other’, ‘No family relationship’}
Type of residence	One of {‘Studio’, ‘Apartment’, ‘House’, ‘Rest home’, ‘Chalet’, ‘Other’}
How many rooms does your residence have^b^?	One of {‘1’, ‘2’, ‘3’, ‘+3’}
Do you have access to any of the following open spaces?	Multiple choice from of {‘Balcony or terrace’, ‘Garden’, ‘Courtyard’, ‘Other’, ‘Any of these’}
Employment status before the start of the crisis	One of {‘Employee’, ‘Self-employed’, ‘Unemployed’, ‘Student’, ‘Retired’, ‘Other’}
Current employment status	One of {‘Not changed’, ‘Teleworking’, ‘Reduced workday’, ‘Increased workday’, ‘Temporary Employment Regulation (ERTE)’, ‘Fired’, ‘New employment’, ‘Other’}
Net monthly income	One of {‘Less than 500€’, ‘500 to 999€’, ‘1000 to 1499€’, ‘1500 to 1999€’, ‘2000 to 2499€’, ’2500 to 2999€’ ‘3000 to 4999€’, ‘5000 to 6999€’, ‘7000 to 8999€’, ‘More than 9000€’}
Do you consider that the crisis has negatively affected your economic situation?	One of {‘Yes’, ‘No’}
Regarding COVID-19	One of {‘I don’t have symptoms’, ‘I have symptoms but have not been diagnosed’, ‘I am diagnosed’}
From the people who live with you	One of {‘No one have symptoms’, ‘One or more have symptoms but have not been diagnosed’, ‘One or more have been diagnosed’}
Before the lockdown, how many hours of physical activity did you get weekly?	One of {‘Less than 2h’, ‘2 to 4h’, ‘4 to 6h’, ‘6 to 8h’, ‘More than 8h’}
Generally, how do you consider your mood before the crisis?	Visual Analogue Scale ranging from ‘Very negative’ to ‘Very positive’

^a^This question is displayed only if the answer to the previous question is one or more. It adds two fields for each family member, one for age and one for relationships. ^b^This question is displayed only if the answer to the previous question is ‘Apartment’ or ‘House’.

**Table 3:** Mood assessment questionnaire.

Question	Response options
How do you feel right now?	Visual Analogue Scale ranging from ‘Very bad’ to ‘Very good’
How physically active do you feel right now?	Visual Analogue Scale ranging from ‘Not active’ to ‘Very active’

**Table 4:** Weekly context questionnaire.

Question	Response options
Have you been diagnosed with COVID-19 during the past week?	One of {‘Yes’, ‘No’}
Has someone living with you been diagnosed with COVID-19 during the past week?	One of {‘Yes’, ‘No’}
Has some relative (not living with you) been diagnosed with COVID-19 during the past week?	One of {‘Yes’, ‘No’}
How do you consider your overall health status?	Visual Analogue Scale ranging from ‘Very bad’ to ‘Very good’
Has your employment status changed during the past week?	One of {‘Not changed’, ‘Teleworking’, ‘Reduced workday’, ‘Increased workday’, ‘Temporary Employment Regulation (ERTE)’, ‘Fired’, ‘New employment’, ‘Other’}
How many hours of physical activity did you get during the past week?	One of {‘Less than 2h’, ‘2 to 4h’, ‘4 to 6h’, ‘6 to 8h’, ‘More than 8h’}
How frequently have you been in contact with relatives, friends and neighbours during the past week?	One of {‘Same as before the lockdown’, ‘Less than before the lockdown’, ‘More than before the lockdown’}
On average, how many hours do you sleep every night?	One of {‘Rather less than usual’, ‘Slightly less than usual’, ‘Same as usual’, ‘Slightly more than usual’, ‘Rather more than usual’}

#### Intake questionnaire

The intake participation questionnaire was a mandatory step during the registration of the participants at the website. The questionnaire was designed to collect data on demographics, residence characteristics, employment, presence of COVID-19 symptoms, and physical and emotional status previous to the confinement. Specifically, the questionnaire gathered the following data: (a) gender, (b) age, (c) postal code, (d) number of house residents, (e) age of the other residents, (f) relationship with the other residents, (g) type of residence, (h) access to open spaces, (i) employment status before the crisis, (j) current employment status, (k) net monthly income, (l) presence of COVID-19 symptoms, (m) presence of COVID-19 symptoms in other residents, (n) hours of physical exercise practice before the crisis, (o) valence assessment before the crisis onset. Detailed information about questions and response options is available in Table 2.

#### Mood assessment questionnaire

Mood variations were monitored with the same methodology regardless of the input method (website or app). Subjective feeling (valence) and physical arousal were assessed using two visual analogue scales (Fig. 3). Valence was evaluated with a modified version of the Feeling Scale^17^ ranging from −50 to +50, including anchors located at −50 = “Very bad” and +50 = “Very good”. Arousal was evaluated with a modified version of the Felt Arousal Scale^18^, ranging from 0 to +100 with anchors provided at 0 = “Not active”, and +100 = “Very active”. The question displayed for valence was: “How do you feel right now?” and for arousal “How physically active do you feel right now?”. For each question the initial slider value was randomly assigned. Detailed information about questions and response options is available in Table 3.

#### Weekly context questionnaire

In order to track possible changes in the contextual and socioeconomic status during the study period, a weekly questionnaire was delivered via the smartphone app. This questionnaire gathered data regarding changes in COVID-19 diagnosis, health status and employment, occurring during the past week. It included the following questions: (a) diagnosis of COVID-19, (b) diagnosis of COVID-19 in other residents, (c) diagnosis of COVID-19 among relatives and close friends, d) current health status, (e) changes in employment status, (f) hours of physical exercise practice during the last week, (g) frequency of social contacts, (h) sleeping patterns. Detailed information about questions and response options is available in Table 4.

### Limitations

The methodology of data collection designed for this study offered participants two channels to report their mood: through the website and through the CoVidAffect smartphone app. The app was developed only for Android OS, and was not available for smartphones with different operating systems. At the time of the study, the mobile devices market share of Android OS in Spain was 78%^19^, meaning that a significant number of participants may not have been able to use their smartphones for data recording. In addition, a preliminary analysis of the data also showed significant differences in valence and arousal between the two data entry modalities (app or web). The average valence of participants who introduced only one mood recording via the web was 2.0 (*std* = 24.5), while the average valence of participants who adhered to the study protocol for two or more days was 9.8 (*std* = 17.6). Differences in arousal were less pronounced with the average arousal of “one-shot” participants being 44.2 (*std* = 25.6) versus 48.4 (*std* = 18.9) for those who repeated mood recordings. These statistically significant differences (*p*_*valence*_ < 0.001; *p*_*arousal*_ = 0.009) may be partly related to the existence of a positive trend found in participants with multiple recordings (average slope of 0.092 in valence and 0.135 in arousal), but could also indicate differences in other aspects of the sample that were not assessed in the study. Finally, the voluntary enrollment to the study introduced a significant selection bias that cautions against the generalisation of any study results to the general population.

## Data Records

Study data, including questionnaire description, associated metadata and documentation, are stored and available at the Zenodo repository^20^, in line with FAIR principles and recent RDA guidelines for COVID-19-related datasets^21^. All data records are also mirrored to the CoVidAffect project at Open Science Framework (OSF)^22^. The data has been stored in three comma-separated values (CSV) files that are described in detail below.

**Fig. 4:**
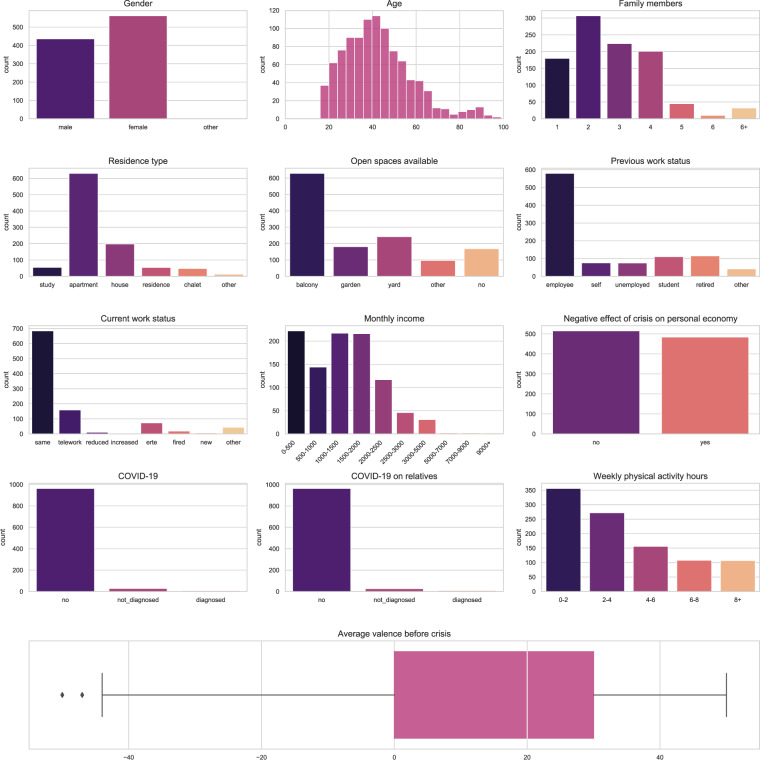
Description of the participant’s sample.

The responses to the intake questionnaire are stored in the file participants.csv. The variables, data types and possible values of this file are described in Table 5. Demographic and baseline characteristics of study participants based on this questionnaire are illustrated in Fig. 4.

**Table 5:** Description of the file participants.csv.

Variable name	Description	Values	Values
id	Unique identifier of the participant	Integer	
registered_date	Timestamp of the participant’s registration at the website	Datetime	YYYY-MM-DD HH:MM:SS
sex	Gender of the participant	String	One of {‘fem’, ‘masc’, ‘other’}
age	Age of the participant	Integer	16 to 100
postcode	Postcode of the participant	Integer	
family_members	Number of people in the residence	String	One of {‘1’, ‘2’,‘3’, ‘4’, ‘5’, ‘6+’}
family_ages	Age of each co-resident specified in the variable family^a^	String	Ordered, comma-separated values for each co-resident from 0 to 100 (e.g. ‘23,34,75’)
family_relation	Relationship between the participant and each co-resident specified in the variable family^a^	String	Ordered, comma-separated values for each co-resident from of {‘parent’, ‘partner’, ‘child’, ‘sibling’, ‘grandparent’, ‘grandchild’, ‘other’, ‘any’} (e.g. ‘partner, sibling’)
residence_type	Type of participant’s residence	String	One of {‘study’, ‘apartment’, ‘house’, ‘residence’, ‘chalet’, ‘other’}
residence_rooms	Number of rooms in the participant’s residence^b^	String	One of {‘1’, ‘2’, ‘3’, ‘3+’}
open_spaces	Open spaces available at the participant’s residence	String	Comma-separated values from of {‘balcony’, ‘garden’, ‘yard’, ‘other’, ‘no’}
work_previous	Employment status of the participant before the crisis	String	One of {‘employee’, ‘self’, ‘unemployed’, ‘student’, ‘retired’, ‘other’}
work_current	Current employment status of the participant	String	One of {‘same’, ‘telework’, ‘reduced’, ‘increased’, ‘erte’, ‘fired’, ‘new’, ‘other’}
income	Net monthly income of the participant coded in 10 possible ranges (in euros)	String	One of {‘0-500’, ‘500–1000’, ‘1000–1500’, ‘1500–2000’, ‘2000–2500’, ‘2500–3000’, ‘3000–5000’, ‘5000–7000’, ‘7000–9000’, ‘9000+’}
negative_economy	Binary flag to indicate the participant’s perception on whether the crisis has negatively affected to his/her economic situation (0=‘no’, 1=‘yes’)}	Integer	One of {0, 1}
covid	Presence of COVID-19 symptoms in the participant	String	One of {‘no’, ‘not_diagnosed’, ‘diagnosed’}
covid_residence	Presence of COVID-19 symptoms in other co-residents	String	One of {‘no’, ‘not_diagnosed’, ‘diagnosed’}}
physical_activity	Number of hours dedicated to physical activity by the participant before the crisis	String	One of {‘0–2’, ‘2–4’, ‘4–6’, ‘6–8’, ‘8+’}
valence	Valence rating of the participant before the crisis	Integer	−50 to 50
input_method	Method used by the participant to report mood records	String	One of {‘App’, ‘Web’, ‘Both’}

^a^This field is empty if *family_members* value is ‘1’ or ‘6+’. ^b^This field is empty unless *type_living* value is ‘apartment’ or ‘house’.

**Fig. 5:**
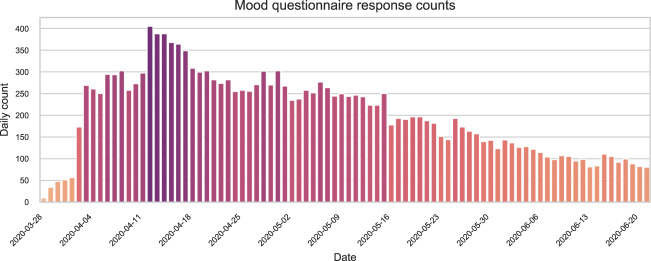
Daily amount of responses to mood questionnaire during study period.

**Fig. 6:**
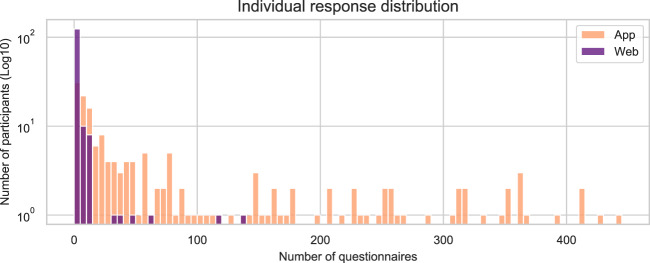
Distributions of Individual responses to mood questionnaires. Responses submitted through the app are represented in orange, and those submitted through the website, in purple. For visualization purposes, participants with only one mood report are not included in this figure.

**Fig. 7:**
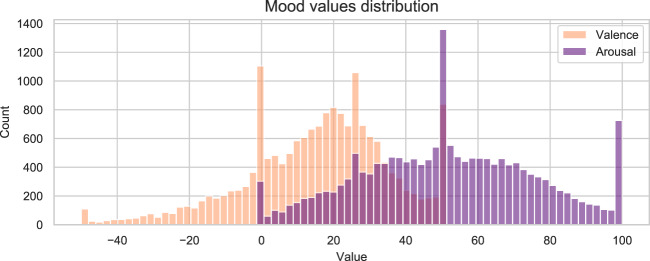
Distribution of reported valence and arousal values.

**Fig. 8:**
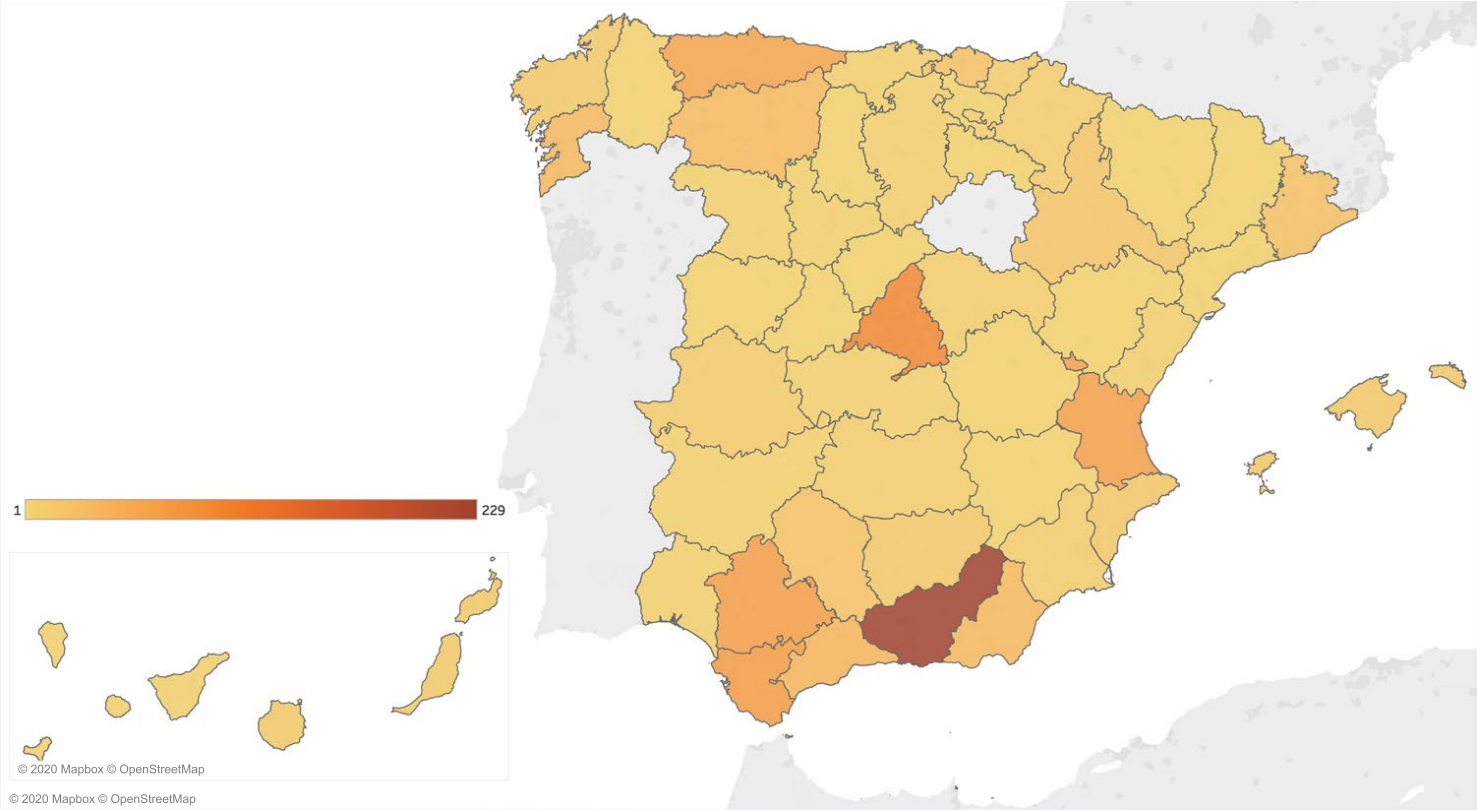
Geographical distribution of the study participants.

The mood ratings registered through the mood questionnaire are stored in the file mood.csv. The variables, data types and possible values of this file are described in Table 6. Note that all records have the same structure regardless of the input method (website or app). The number of mood questionnaire responses submitted each study day is illustrated in Fig. 5. The distributions of the total amount of mood questionnaire responses provided by one user are depicted in Fig. 6. The distribution of the valence and arousal values reported is shown in Fig. 7. Finally, the geographical distribution of the participants within the Spanish territory is illustrated in Fig. 8. The three provinces with the largest number of participants are Granada, Madrid and Cádiz.

**Table 6:** Description of the file mood.csv.

Variable name	Description	Type	Values
participant	Unique identifier of the participant (*id* variable of *participants.csv*)	Integer	
timestamp	Timestamp on which the questionnaire notification was triggered by the smartphone app	Datetime	YYYY-MM-DD HH:MM:SS
answer_timestamp	Timestamp of the mood report	Datetime	YYYY-MM-DD HH:MM:SS
valence	Current valence rating	Integer	−50 to 50
arousal	Current arousal rating	Integer	0 to 100
valence_scale_ini	Starting position of *valence* input slider	Integer	−50 to 50
arousal_scale_ini	Starting position of *arousal* input slider	Integer	0 to 100
input_method	Method used by the participant to report the mood record	String	One of {‘App’, ‘Web’, ‘Both’}

In the case mood was reported through the website, the same value is assigned to the variables *timestamp* and *answer_timestamp*.

The responses to the context questionnaire are stored in the file context.csv. The variables, data types and possible values of this file are described in Table 7. Note that these data are only recorded by participants using the smartphone app.

**Table 7:** Description of the file context.csv.

Variable name	Description	Type	Values
date	Date on which the questionnaire was answered (always on Friday at 20:00h, Madrid timezone)	Date	DD/MM/YYYY
participant	Unique identifier of the participant (corresponding to the *id* variable of *participants.csv*)	Integer	
covid_diagnosed	Binary flag to indicate whether the participant has been diagnosed with COVID-19 during the past week	String	One of {‘Yes’, ‘No’}
covid_residence_diagnosed	Binary flag to indicate whether any coresident was diagnosed with COVID-19 during the past week	String	One of {‘Yes’, ‘No’}
covid_family_diagnosed	Binary flag to indicate whether any family member or close person was diagnosed with COVID-19 during the past week	String	One of {‘Yes’, ‘No’}
perceived_health	Participant’s perception on his/her general health status during the past week	Integer	1 to 5
work_changed	Changes in work status during the past week	String	One of {‘no’, ‘telework’, ‘reduce’, ‘increase’, ‘erte’, ‘fired’, ‘new’, ‘other’}
physical_activity	Number of hours dedicated to physical activity during the past week	String	One of {‘0–2’, ‘2–4’, ‘4–6’, ‘6–8’, ‘8+’}
social_contact	Social contact frequency during the past week compared to social contact before the crisis	String	One of {‘same’, ‘less’, ‘more’}
sleep	Sleep quantity during the past week, compared to the average before the crisis	String	One of {‘lot_less’, ‘less’, ‘same’, ‘more’, ‘lot_more’}

## Technical Validation

To ensure data reliability, participants who submitted mood data through the website were required to insert their identification number and postal code, which were checked against the existing registers in the database. This step was not required when entering data through the smartphone app.

The visual analogue scales used to rate both valence and arousal were randomly initialized to avoid any bias caused by selection effort. Also, the mood assessment and weekly context questionnaires triggered through the smartphone app only showed one question per page. This technique helped the participant to focus on one question at a time.

The database was periodically checked to identify possible duplicate records. We considered answers by the same participant with the same notification launch timestamp as duplicates and kept only the first register.

## Usage Notes

The dataset, metadata and documentation are publicly available for research purposes at our Zenodo^20^ and OSF^22^ repositories. No request is required for data download. Columns in each CSV file are delimited by semicolons. The responses to multiple choice questions from the initial questionnaire (see Tables 2 and 5) have been encoded using commas to separate the multiple values (e.g. *family_ages* = ‘*12,34,68*’).
